# Oral Mycobiome Analysis of HIV-Infected Patients: Identification of *Pichia* as an Antagonist of Opportunistic Fungi

**DOI:** 10.1371/journal.ppat.1003996

**Published:** 2014-03-13

**Authors:** Pranab K. Mukherjee, Jyotsna Chandra, Mauricio Retuerto, Masoumeh Sikaroodi, Robert E. Brown, Richard Jurevic, Robert A. Salata, Michael M. Lederman, Patrick M. Gillevet, Mahmoud A. Ghannoum

**Affiliations:** 1 OHARA/ACTG Mycology Unit at Case Western Reserve University, Department of Dermatology, Cleveland, Ohio, United States of America; 2 Center for Medical Microbiology, Department of Dermatology, School of Medicine, Case Western Reserve University and University Hospitals Case Medical Center, Cleveland, Ohio, United States of America; 3 Microbiome Analysis Center, Department of Environmental Science and Policy, George Mason University, Fairfax, Virginia, United States of America; 4 Department of Biological Sciences, School of Dental Medicine, Case Western Reserve University, Cleveland, Ohio, United States of America; 5 Division of Infectious Diseases and HIV Medicine, Case Western Reserve University, University Hospitals Case Medical Center, Cleveland, Ohio, United States of America; Geisel School of Medicine at Dartmouth, United States of America

## Abstract

Oral microbiota contribute to health and disease, and their disruption may influence the course of oral diseases. Here, we used pyrosequencing to characterize the oral bacteriome and mycobiome of 12 HIV-infected patients and matched 12 uninfected controls. The number of bacterial and fungal genera in individuals ranged between 8–14 and 1–9, among uninfected and HIV-infected participants, respectively. The core oral bacteriome (COB) comprised 14 genera, of which 13 were common between the two groups. In contrast, the core oral mycobiome (COM) differed between HIV-infected and uninfected individuals, with *Candida* being the predominant fungus in both groups. Among *Candida* species, *C. albicans* was the most common (58% in uninfected and 83% in HIV-infected participants). Furthermore, 15 and 12 bacteria-fungi pairs were correlated significantly within uninfected and HIV-infected groups, respectively. Increase in *Candida* colonization was associated with a concomitant decrease in the abundance of *Pichia*, suggesting antagonism. We found that *Pichia* spent medium (PSM) inhibited growth of *Candida*, *Aspergillus* and *Fusarium*. Moreover, *Pichia* cells and PSM inhibited *Candida* biofilms (*P* = .002 and .02, respectively, compared to untreated controls). The mechanism by which *Pichia* inhibited *Candida* involved nutrient limitation, and modulation of growth and virulence factors. Finally, in an experimental murine model of oral candidiasis, we demonstrated that mice treated with PSM exhibited significantly lower infection score (*P* = .011) and fungal burden (*P* = .04) compared to untreated mice. Moreover, tongues of PSM-treated mice had few hyphae and intact epithelium, while vehicle- and nystatin-treated mice exhibited extensive fungal invasion of tissue with epithelial disruption. These results showed that PSM was efficacious against oral candidiasis *in vitro* and *in vivo*. The inhibitory activity of PSM was associated with secretory protein/s. Our findings provide the first evidence of interaction among members of the oral mycobiota, and identifies a potential novel antifungal.

## Author Summary

Oral microbiota contribute to health and disease, and their disruption may influence the course of oral diseases like oral candidiasis. Here we identify the core oral mycobiome (COM) and core oral bacteriome (COB) in HIV-infected and uninfected individuals, and demonstrate that the COM differs between these two groups. Decrease in abundance of *Pichia* (a resident oral fungus) in uninfected individuals coincided with increase in abundance of *Candida*, suggesting an antagonistic relationship. In vitro testing showed that *Pichia* spent medium (PSM) inhibits growth of pathogenic fungi; these findings were validated in an experimental mouse modal of oral candidiasis. The mechanism by which *Pichia* antagonizes *Candida* involves nutrient competition and secretory factor/s that inhibit the latter's ability to adhere, germinate, and form biofilms. This study is the first to characterize the mycobiome and the bacteriome in the oral cavity of HIV infected patients, and provides the first evidence that a fungus present in the same host microenvironment antagonizes *Candida* and identifies potential novel antifungal approach.

## Introduction

Organisms residing in the oral cavity (oral microbiota) contribute to health and disease, and influence diseases like oral candidiasis, the most common oral complication of HIV-infection [1], [2]. Pathogenesis of oral candidiasis is linked to variables like changes in the CD4+ cell count and antiretroviral therapy (ART) in HIV-1-infected patients [3]. Although the introduction of ART has reduced mortality and morbidity as well as the incidence of opportunistic infections among HIV-infected patients, oral candidiasis remains a significant disease, even in the era of ART. In this regard, recent studies indicate that the decline of oral candidiasis among ART-experienced HIV-infected patients is transient in some HIV-infected individuals [4]. In addition, preliminary results reported by Thompson et al. [5] showed that symptomatic oral *Candida* infection occurred in one-third of patients with advanced AIDS (n = 122), even in the setting of ART. More recently, Patel et al. [6] reported symptomatic oral candidiasis in 27% (59/215) HIV-infected patients. Therefore, even in the era of ART, oral candidiasis remains a significant problem.

Characterization of the microbiota (bacteriome and mycobiome) in health and disease is expected to expedite the discovery, testing and validation of novel drugs [7]. Most studies that characterized the human microbiome in health and disease have focused on the bacteriome, in both oral and non-oral body sites [8]–[12]. Recently, Iliev *et. al.* [13] showed that while no significant differences in major phyla of commensal bacteria was observed in the intestinal microflora between wild-type and mice lacking Dectin-1, members of the mycobiome interacted with the intestinal immune system to influence inflammatory bowel disease (IBD) highlighting the role of the fungal community in disease. Earlier, we characterized the oral mycobiome in healthy individuals using high-throughput multitag pyrosequencing (MTPS), and reported that humans are colonized with up to 85 fungal genera [14]. Although these studies demonstrated the complexity of the human oral microbiome, the specific contribution of the mycobiome to oral diseases was not investigated.

Previous studies have shown that alteration in the bacterial population has direct impact on the development of *Candida* infections [for reviews, see 15]. However, the interactions between members of the oral microbiota and *Candida* in HIV disease setting have not been investigated. In the current study, we identified the core oral mycobiome (COM) and bacteriome (COB) [defined as those organisms present in ≥20% of the subjects] in HIV-infected and uninfected individuals, and demonstrated that the COM undergoes a change in HIV disease. Furthermore, we noted that a decrease in abundance of the yeast *Pichia* coincided with an increase in *Candida* colonization, suggesting an antagonistic relation between these two fungi. We also found that nutrient competition as well as *Candida* growth and modulation of its virulence factors by *Pichia* is a mechanism underlying this interaction. In addition, treatment with *Pichia* Spent Medium (PSM) was efficacious against oral candidiasis when tested in an experimental murine model. Our results provide the first evidence of interaction among members of the oral mycobiome community, particularly between *Pichia* and pathogenic fungi. These findings could lead to the development of novel antifungals to prevent and treat fungal infections including mucosal *Candida* infections, thereby impacting the management of oral candidiasis and other fungal infections. They also highlight the need to characterize the mycobiome at different body sites which may lead to novel discoveries of how fungi can be exploited to control health and disease.

## Materials and Methods

### Ethics Statement

Written informed consent was obtained from all study participants. Study participants were recruited according to protocol (#20070413) approved by the Human Subjects Institutional Review Board (IRB) at University Hospitals Case Medical Center. Oral rinse samples were obtained from 12 HIV-infected and 12 uninfected individuals (matched for age, sex, and ethnicity). Inclusion criteria for these participants were: >18 years of age and no clinical signs of oral mucosal disease including oral candidiasis, while the exclusion criteria were: recent use of antimicrobial or antifungal agents (within a month), use of topical or systemic steroids, pregnancy, and insulin-dependent diabetes mellitus. Oral wash samples were collected using a standardized Standard Operating Procedure developed by the Oral HIV/AIDS Research Alliance (OHARA) as described earlier [14]. All animal experimentation was performed in strict accordance with the Guide for the Care and Use of Laboratory Animals of the National Institutes of Health. The protocol for animal infection was approved by the Institutional Animal Care and Use Committee (IACUC) at Case Western Reserve University School of Medicine, Cleveland, Ohio (protocol approval number 2013-0020). All procedures were performed under general anesthesia and all efforts were made to minimize animal suffering.

### Microbiome Analysis

Oral rinse samples were processed individually using the Fast DNA Spin Kit following manufacturer's instructions (BIO 101; Vista, CA). Each extraction tube was agitated three times using a Fast Prep FP120 instrument at a speed setting of 5 for 30 s. Tubes were cooled on ice between agitations. Fungi and bacteria present in these samples were identified with ITS-based and 16S probes, respectively. The ITS1 region from DNA sample extracts was amplified in triplicate using primers with high specificity for ascomycete fungi (fluorescently labeled forward primer ITS1F (CTTGGTCATTTAGAGGAAGTAA) and unlabeled reverse primer ITS2 (GCTGCGTTCTTCATCGATGC). The ITS primers were selected in this study to detect the presence of various fungi since these primers are able to detect consensus sequences present in a broad range of fungi [16], [17]. For bacterial identification, extracted DNA was amplified by PCR using routinely employed universal primers [fluorescently labeled forward primer 27F (5′-6FAM- AGAGTTTGATCCTGGCTCAG-3′) and unlabeled reverse primer 355R5′ (5′- GCTGCCTCCCGTAGGAGT-3′)] [18], which amplify the first two hyper-variable regions of 16S rRNA [19] and are commonly used for microbiome analysis [10], [20]. Microbiome analysis was performed using multitag 454 pyrosequencing (MTPS) technique, which was used for characterization of nucleic acids [21] (for details, please see Method S1).

### Strains

*Candida albicans* [strains SC5312, 10341, GDH2346), *Pichia* (MRL81)], *Penicillium* (MRL22345) and *Cladosporium* (MRL1458) strains tested in this study were obtained from the OHARA Repository at Case and the culture collection of the Center for Medical Mycology. Fungal strains were maintained on Sabouraud dextrose agar (SDA, [yeast extract, peptone, and dextrose at 1∶2∶1]) (Difco Laboratories, Detroit, MI) medium. Since species-level identification of *Pichia* based upon morphological or physiological features alone is usually not possible, we used a molecular approach (based on sequence analysis of the internal transcribed spacer and D1/D2 ribosomal DNA regions) [22] to confirm the identity of *Pichia* MRL81 strain. Our analysis revealed that this strain was *P. farinosa*. All strains were kept at −80°C for long-term storage.

### Role of Nutrient Limitation

To determine whether inhibition of pathogenic fungi by *Pichia* spent medium (PSM) is due to nutrient competition between *Pichia* and *Candida*, we assessed *Candida* growth when mixed with *Pichia* at different ratios. *P. farinosa* and a GFP-tagged *C. albicans* strain (generous gift from Dr. B. Cormack) [23] were mixed together at equivalent cell densities (10^3^, 10^4^, or 10^5^ cells each) in yeast nitrogen base (YNB) medium supplemented with glucose (0.9% w/v) and uridine (100 µg/mL). These mixed cell suspensions were incubated at 37°C and allowed to grow (as “mixed cultures”). *Candida* or *Pichia* cells alone were grown in the same growth medium (“mono cultures”) serving as controls. At specific time intervals (24, 48, 72 h), 1-mL aliquots were withdrawn from the mixed- and mono-cultures, centrifuged to collect the cells in pellet, and re-suspended in 250 µL phosphate buffered saline. Fifty µL of the resuspended cell mixture was placed on a glass slide and observed under phase-contrast and fluorescence microscope. The remaining cell suspension (200 µL) was transferred to a 96-well microtiter plate, and fluorescence was measured using a spectrofluorimeter (excitation and emission wavelength of 485 and 435 nm, respectively).

### Biochemical Characterization of *Pichia* Spent Medium (PSM)

The effect of *Pichia* supernatant on *Candida* growth, germination, adherence, biofilm formation, and its biochemical properties was determined as described below.

### Effect of *Pichia* Supernatant on the Growth of *Candida*, *Aspergillus*, and *Fusarium*

To evaluate the effect of *Pichia* supernatant on the growth of various fungi, *Pichia* spent (“conditioned”) medium (PSM) was obtained by centrifuging 100-mL culture of *Pichia* grown in Sabouraud dextrose broth (SDB) for 48 h, and filter sterilizing it. Next, fungal cells/conidia (1×10^5^ cells/mL) were incubated with PSM at 35°C and growth was followed for 48 h. Aliquots were collected at 2 h intervals and fungal growth was measured spectrophotometrically at 600 nm.

### Germination Assay

Effect of *Pichia* spent media (PSM) on *Candida* germination was determined using *C. albicans* strain SC5314, as described previously [24]. *Candida* cells were grown planktonically in the absence or presence of PSM. Germination rate was compared with that of cells grown in SDB media containing fetal bovine serum (FBS) (Hyclone, Thermo Fisher Scientific, Rockford, IL), a known inducer of germination. Briefly, *C. albicans* cells were grown and the cell density was adjusted to 1×10^7^ cells/mL in Hanks balanced salt solution (HBSS) (Mediatech). In separate 1.5-mL tubes, 50 mL of these cells were diluted to a density of 5×10^5^ cells/mL in HBSS (blank control), FBS, or PSM, and incubated on a rocker at 37°C for up to 4 h. At 15-min intervals, 10-µL samples from each media type were microscopically examined using a hemacytometer. Total cell count and germination (defined as a germ-tube length greater than or equal to the blastospore diameter) was determined from an average of 4 observations. One hundred to 200 cells were counted per observation. The assays were discontinued when cells clumped together, due to germination, which made it difficult to count individual cells.

### Adhesion Assay

The effect of *Pichia* or *Penicillium* (used as a control) cells or supernatant on *Candida* adherence (using strain *C. albicans* SC5314, a clinical isolate used conventionally in *Candida* adhesion and germination assays) was determined as described earlier [24], [25]. Briefly, standardized suspensions of 50 to 200 cells/mL were added onto silicone elastomer disks for 90 min. Disks were then washed in phosphate-buffered saline (PBS) to remove non-adherent cells and placed in wells of 12-well tissue culture plates (Becton Dickinson, Franklin Lakes, NJ). Two milliliters of warm (55°C) liquefied SDA was added per well to completely cover the SE disks and allowed to solidify. Plates were incubated overnight (37°C), and the number of colonies adhering per disk was counted using a dissecting microscope.

### Biofilm Evaluation

The effect of oral fungi (*Pichia, Cladosporium* or *Penicillium*, the latter fungi were used as controls since *Cladosporium* was present only in the uninfected subjects like *Pichia*, while *Penicillium* was present in both infected and uninfected subjects to the same abundance) or their supernatant on the ability of *Candida* to form biofilms was evaluated using metabolic activity assay and confocal microscopy, as described earlier [26]–[28]. Briefly, *Candida* cells were incubated in the presence or absence of *Pichia* cells or spent medium (supernatant, PSM) at different relative ratios (1∶3, 1∶1, 3∶1), and allowed to form biofilms for 48 h on silicone elastomer catheter discs. The amount of biofilm formed was assayed colorimetrically using the XTT (2,3-bis (2-methoxy-4-nitro-5-sulfophenyl)-5-[(phenylamino) carbonyl]-2H-tetrazolium hydroxide, Sigma-Aldrich) metabolic activity assay in which XTT is converted by metabolically active cells to a red formazan product [26]. In addition, the effect of fungal supernatants on the morphology and architecture of the formed biofilms was evaluated using confocal scanning laser microscopy (CSLM) [26]. Briefly, biofilms were stained with the fluorescently labeled polysaccharide-indicating lectin Concanavalin Alexa Fluor 488 conjugate (CON-A, 25 µg/mL; Invitrogen) and metabolic activity indicator dye FUN1™ (10 µM; Invitrogen). After staining, discs containing biofilms were flipped and placed on a 35-mm-diameter glass-bottom petri dish (MatTek Corp., Ashland, Mass.). Stained biofilms were observed with a Zeiss LSM510 confocal scanning laser microscope equipped with argon and HeNe lasers and mounted on a Zeiss Axiovert100 M microscope (Carl Zeis, Inc.). The objective used was a water immersion C-apochromat lens (40×; numerical aperture, 1.2).

### *In Vivo* Model of Oral Candidiasis

Wild-type C57BL/6 mice (purchased from Charles River Laboratories, Wilmington, MA) were immunosuppressed with 4 mg of cortisone acetate (Sigma Chemical Co., St. Louis, Mo.) administered subcutaneously on the day before and 1 and 3 days after challenge with *Candida* cells. Mice were given tetracycline hydrochloride (Sigma Chemical Co., St. Louis, Mo.) in their drinking water (0.5 mg/ml), starting the day before infection. On the day of inoculation, mice were anesthetized and light scratches made on the dorsum of the tongue followed by the introduction of *C. albicans* GDH (10^8^ blastospores). The scratches were superficial, limited to the outermost stratum corneum, and did not cause trauma or bleeding. Mice were divided into groups (n = 4); treated with *Pichia* supernatant, 100 µl in the oral cavity twice a day, a “mock” vehicle control, and untreated control. Topical nystatin (widely used clinically to treat oral candidiasis [29]) was used as a comparator. Treatment began on day 4 post inoculation, mice were sacrificed on day 7 and the tongues harvested for enumeration of tissue fungal burden or histopathology with Periodic acid-Schiff stain. Additionally, tongues were visually assessed daily beginning day 1 post infections to assess severity of the infection using a previously described scoring system [30]: A score of 0 indicates the appearance of a normal tongue, with intact light reflection and no visible signs of infection, a score of 1 denotes isolated patches of fungus, a score of 2 when confluent patches of fungus are observed throughout the oral cavity, and a score of 3 indicates the presence of wide-spread fungal plaques and erosive mucosal lesions. The histology slides were assessed by an independent pathologist who was blinded to the study arms. The animal studies were repeated on different days to ensure reproducibility.

### Identification of PSM as a Protein

Since the antifungal activity of PSM was secretory in nature, we determined whether this activity was due to a protein, carbohydrate or small molecule (metabolite). We exposed PSM to proteinase K (which digests most proteins), NaOH (which denatures carbohydrates) [31], or acetonitrile extraction (that isolates metabolites) [32]. We also determined the effect of heat on PSM activity by exposing it to 90°C temperature in a water bath for 10 min. The ability of these differently treated PSM to inhibit *Candida* biofilms was evaluated as above.

### Statistical Analysis

Microbiome data were analyzed using the QIIME and R platforms [33], [34]. Wilcoxon–Mann–Whitney rank sum test was used for comparison between the two groups, with *P*-value of <0.05 considered as a significant difference. For comparison of several groups, Kruskal–Wallis one-way analysis of variance on ranks was used, and pairwise multiple comparison procedures (Dunns method) post-hoc test was used for multiple pairwise comparisons. Univariate analyses was used to compare the prevalence of specific fungi and bacteria between the two groups using a Pearson chi-squared test or two-sample t-test (assuming unequal variances). For correlation analysis, microbiome abundance data was divided into independent data matrices (disease and no disease) and correlation analyses was conducted using the “psych” package (corr.test function) in R statistical platform (pairwise Spearman's correlation and two-tailed probability of *t* for each correlation) [34], [35]. The function “Circle.corr” was used to graphically illustrate the correlation coefficients and significant correlations (*P*<.05) in circle graph, where red and blue circle indicate positive and negative correlations, respectively. Numerical variables (e.g. metabolic activity, dry biomass, thickness) were all assessed using paired or unpaired t-test, or ANOVA as appropriate. Comparison of clinical scores were performed using box-plots and non-parametric independent samples Kruskal-Wallis test, while mean fungal burden (log CFU/g) were compared using ANOVA. All statistical analyses were performed using R [34] or SPSS (ver. 13) statistical software packages.

## Results

### Participant Demographics

A total of 24 individuals were enrolled in the study, with 12 HIV-infected patients and 12 uninfected individuals (11 males and one female in both study groups, Table 1). The mean age was 38.7 and 38.8 years in HIV-infected (age range: 22–56) and uninfected (age range: 22–59) groups, respectively. Among the 12 HIV-infected patients, eight had initiated antiretroviral therapy. In both study groups, self-reported ethnicities were: six African-Americans, two Hispanics, and four Caucasians. While all samples were analyzed for fungal microbiota, one of the samples did not provide robust signals for the bacterial microbiome, and hence was excluded from the analysis. In addition, the corresponding matched uninfected control sample was also excluded. As a result, there were 12 uninfected-HIV-infected sample pairs for mycobiome analysis but only 11 sample pairs for bacteriome analysis.

**Table 1:** Demographic information of study participants.

Group	ID	Age (years)	Gender§	Ethnicity*	CD4 cell count (cells/mm^3^)	Viral load (U/mL)	Medication
HIV-infected	1	31	M	H (W)	380	158000	None
	2	56	M	AA	639	75	Atripla
	3	52	M	AA	800	48	Atripla
	4	40	M	C (W)	947	48	Atripla
	5	40	M	C (W)	280	48	Atripla
	6	22	M	AA	966	1100	None
	7	31	F	H (W)	1029	48	Rotanivir, Fosamprenavir, Combivir
	8	42	M	AA	814	53	Rotanivir, Atazanavir, Truvada
	9	22	M	C (W)	581	115000	None
	10	52	M	AA	5	185000	None
	11	31	M	C (W)	670	68	Atripla
	12	45	M	AA	899	48	Rotanivir, Atazanavir, Truvada
Uninfected	1C	34	M	H (W)	NA	NA	None
	2C	46	M	C (W)	NA	NA	None
	3C	59	M	AA	NA	NA	None
	4C	22	M	C (W)	NA	NA	None
	5C	37	M	AA	NA	NA	None
	6C	34	F	H (W)	NA	NA	None
	7C	40	M	C (W)	NA	NA	None
	8C	27	M	C (W)	NA	NA	None
	9C	53	M	AA	NA	NA	None
	10C	44	M	AA	NA	NA	None
	11C	22	M	AA	NA	NA	None
	12C	47	M	AA	NA	NA	None

§ Gender: M – Male, F – Female.

*Self-reported ethnicity: W = White; H = Hispanic; C = Caucasian; AA = African-American.

### Oral Bacteriome of HIV-infected Participants Were Similar to That of Uninfected Individuals

**Figure 1:**
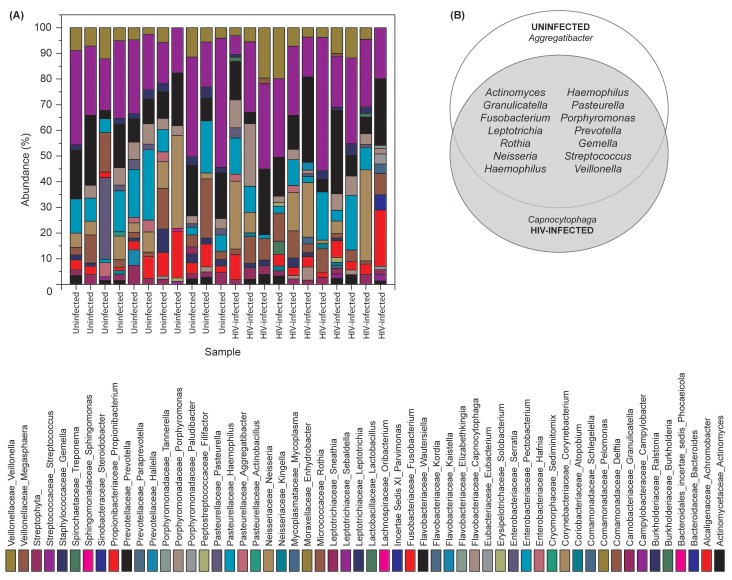
Bacterial microbiome (bacteriome) of HIV-infected patients and uninfected individuals. (A) Relative abundance bacteria in uninfected and HIV-infected patients (N = 11 for each group), (B) Bacteria present in the core oral bacteriome of uninfected and HIV-infected individuals.

Our results showed that the number of bacterial genera in the oral microbiota of study participants ranged between 8–14 per person among HIV-infected and uninfected individuals. Among HIV-infected patients, *Prevotella*, *Streptococcus* and *Rothia* were the most common genera; while in controls the most abundant bacteria were *Prevotella*, *Streptococcus* and *Fusobacterium* (Fig. 1A, and Table S1). The core oral bacteriome (COB) consisted of 14 genera in both HIV-infected and uninfected individuals, of which 13 (*Actinomyces*, *Granulicatella*, *Fusobacterium*, *Leptotrichia*, *Rothia*, *Neisseria*, *Haemophilus*, *Pasteurella*, *Porphyromonas*, *Prevotella*, *Gemella*, *Streptococcus*, and *Veillonella*) were common to both groups (Fig. 1B). We found that *Capnocytophaga* was present only in HIV-infected patients while *Aggregatibacter* was present in uninfected individuals only (Fig. 1B). These results suggest that the COB of HIV-infected patients was similar to that of uninfected individuals with minimal difference.

### Oral Mycobiome of HIV-infected Patients Exhibits Differences from Uninfected Individuals

**Figure 2:**
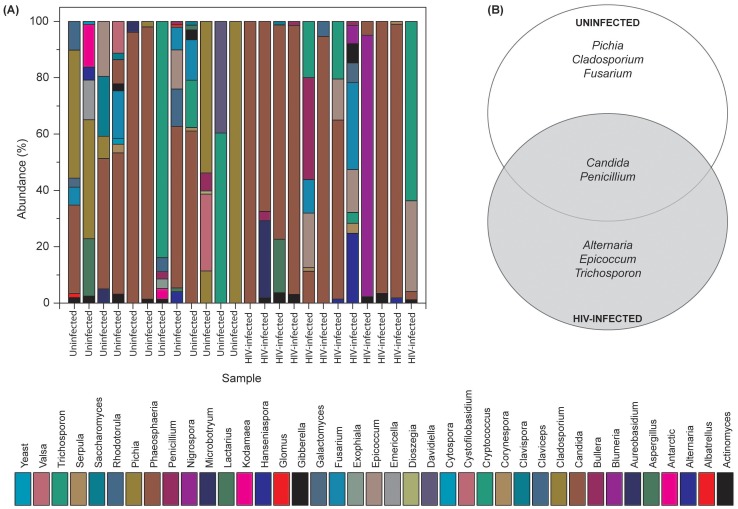
Fungal microbiome (mycobiome) of HIV-infected patients and uninfected individuals. (A) Relative abundance of fungi in uninfected and HIV-infected patients (N = 12 for each group), (B) Fungi present in the core oral mycobiome of uninfected and HIV-infected individuals.

Our results showed that the number of fungal genera present in oral wash samples ranged between 1–9 per person among uninfected and HIV-infected individuals (Fig. 2A and Table S2). Among HIV-infected patients, *Candida*, *Epicoccum*, and *Alternaria* were the most common genera (present in 92%, 33%, and 25%, respectively), while in uninfected participants, the most abundant fungi were *Candida*, *Pichia*, and *Fusarium* (58%, 33%, and 33%, respectively; Fig. 2A). The COM of HIV-infected and uninfected individuals consisted of five genera (Fig. 2B); of these, *Candida* and *Penicillium* were common between the two groups, while differing in the remaining genera demonstrating that the COM of HIV-infected patients differs from that of age- and sex-matched uninfected controls. Among the *Candida* species detected, *C. albicans* was the most common (58% in uninfected and 83% in HIV-infected patients), followed by *C. dubliniensis* (17% in both groups). Interestingly, *C. intermedia* and *C. sake* were present only in uninfected (n = 1) and HIV-infected (n = 1) groups, respectively.

### Correlation between Members of the Oral Bacteriome and Mycobiome in HIV-Infected Patients

**Figure 3:**
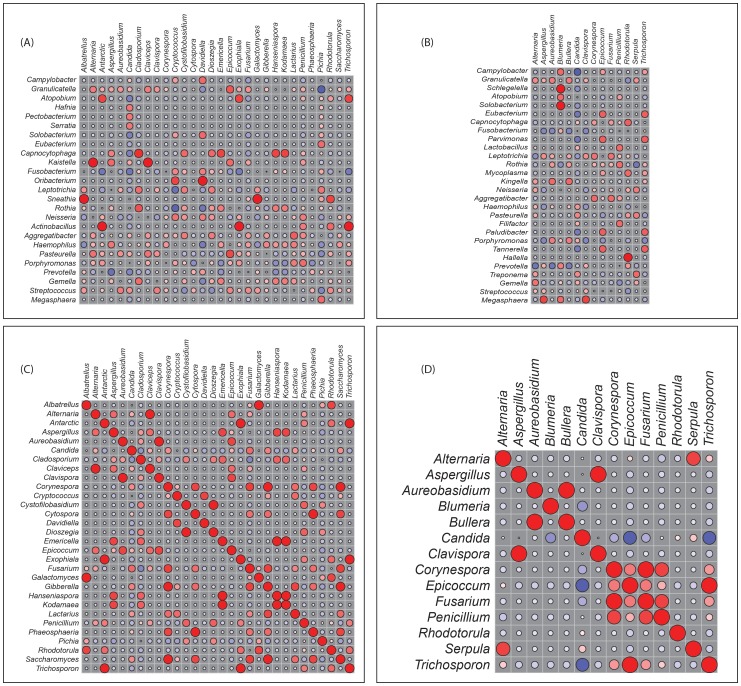
Correlation coefficients of abundance of microbes in uninfected and HIV-infected patients. Correlation of bacteriome and mycobiome was determined for (A) uninfected and (B) HIV-infected study participants using R statistical computing software (Spearman's correlation and two-tailed probability of *t* for each correlation) for the two groups. Interactions among different fungi in the mycobiome of (C) uninfected and (D) HIV-infected study participants was also determined using similar approach. Red: Positive correlation; Blue: negative correlation; diameter of circles represent the absolute value of correlation for each pair of the microbe-microbe matrix.

Next, we determined how the individual members of the oral bacteriome and mycobiome are correlated within their respective communities, and also across the two communities. We grouped the microbiome abundance data into independent mycobiome and bacteriome data matrices and conducted correlation analysis using R statistical computing software. We found 15 bacteria-fungi pairs that were correlated significantly in samples from non-infected study participants (Fig. 3 A, Table 2). Among these significant correlation pairs, two pairs (*Rothia*-*Cladosporium* and *Granulicatella*-*Cryptococcus*) were negatively correlated (coefficient −0.61 and −0.65, respectively). The remaining 13 pairs of significantly correlated pairs exhibited positive correlation with coefficients ranging from 0.64 (*Aggregatibacter*-*Lactarius*) to 0.86 (*Capnocytophaga*-*Cladosporium*). In comparison, there were 12 statistically significant bacteria-fungi pairs in HIV-infected patients, with 11 positive (coefficient of 0.64 for 8 pairs, 0.74 for 2 pairs, Fig. 3 B, Table 2) and one with negative correlation (*Campylobacter*-*Candida*, coefficient −0.67).

**Table 2:** Correlation between bacteriome and mycobiome in uninfected and HIV-infected study participants.

Uninfected Individuals				HIV-Infected Patients			
Bacteria	Fungi	P-value	Correlation	Bacteria	Fungi	P-value	Correlation
*Atopobium*	*Antarctic*	0.009	0.74	*Megasphaera*	*Aspergillus*	0.009	0.74
*Capnocytophaga*	*Cladosporium*	0.001	0.86	*Campylobacter*	*Candida*	0.023	−0.67
*Rothia*	*Cladosporium*	0.021	0.68	*Megasphaera*	*Clavispora*	0.009	0.74
*Oribacterium*	*Cryptococcus*	0.009	0.74	*Eubacterium*	*Epicoccum*	0.035	0.64
*Rothia*	*Cryptococcus*	0.048	−0.61	*Parvimonas*	*Epicoccum*	0.035	0.64
*Capnocytophaga*	*Emericella*	0.009	0.74	*Paludibacter*	*Epicoccum*	0.035	0.64
*Granulicatella*	*Epicoccum*	0.025	0.67	*Tannerella*	*Epicoccum*	0.035	0.64
*Pasteurella*	*Epicoccum*	0.012	0.72	*Capnocytophaga*	*Rhodotorula*	0.035	0.64
*Atopobium*	*Exophiala*	0.009	0.74	*Eubacterium*	*Trichosporon*	0.035	0.64
*Capnocytophaga*	*Hanseniaspora*	0.009	0.74	*Parvimonas*	*Trichosporon*	0.035	0.64
*Capnocytophaga*	*Kodamaea*	0.009	0.74	*Paludibacter*	*Trichosporon*	0.035	0.64
*Aggregatibacter*	*Lactarius*	0.035	0.64	*Tannerella*	*Trichosporon*	0.035	0.64
*Granulicatella*	*Pichia*	0.031	−0.65				
*Sneathia*	*Rhodotorula*	0.009	0.74				
*Atopobium*	*Trichosporon*	0.009	0.74				

For correlation analysis, microbiome abundance data was divided into independent data matrices (disease and no disease) and correlation analyses was conducted using the “psych” package (corr.test function) in R statistical platform (pairwise Spearman's correlation and two-tailed probability of *t* for each correlation) [34], [35].

We also evaluated the correlation between different members of the mycobiome in the uninfected and HIV-infected groups. Our analyses revealed that in the uninfected group, 23 fungal-fungal interactions were statistically significant (*P*≤0.035, Fig. 3C, Table 3), while in the HIV-infected group, 6 fungus-fungus pairs were significantly correlated (*P*-value of ≤0.03, Fig. 3D), which included *Candida*-*Epicoccum*, *Candida*-*Trichosporon*, *Epicoccum*-*Trichosporon*, *Penicillium*-*Corynespora*, *Penicillium*-*Fusarium*, and *Alternaria*-*Serpula*.

**Table 3:** Correlation among oral fungi in uninfected study participants.

Fungi	Fungi	*P*-Value	Correlation
*Corynespora*	*Gibberella*	0.001	0.98
*Gibberella*	*Saccharomyces*	0.001	0.98
*Cladosporium*	*Emericella*	0.035	0.64
*Cladosporium*	*Hanseniaspora*	0.035	0.64
*Cladosporium*	*Kodamaea*	0.035	0.64
*Cystofilobasidium*	*Penicillium*	0.035	0.64
*Dioszegia*	*Penicillium*	0.035	0.64
*Albatrellus*	*Rhodotorula*	0.009	0.74
*Aspergillus*	*Emericella*	0.009	0.74
*Aspergillus*	*Hanseniaspora*	0.009	0.74
*Aspergillus*	*Kodamaea*	0.009	0.74
*Aureobasidium*	*Epicoccum*	0.009	0.74
*Clavispora*	*Epicoccum*	0.009	0.74
*Corynespora*	*Cytospora*	0.009	0.74
*Corynespora*	*Phaeosphaeria*	0.009	0.74
*Cryptococcus*	*Davidiella*	0.009	0.74
*Cytospora*	*Saccharomyces*	0.009	0.74
*Galactomyces*	*Rhodotorula*	0.009	0.74
*Gibberella*	*Lactarius*	0.009	0.74
*Phaeosphaeria*	*Saccharomyces*	0.009	0.74
*Fusarium*	*Gibberella*	0.006	0.77
*Corynespora*	*Fusarium*	0.005	0.78
*Fusarium*	*Saccharomyces*	0.005	0.78

### *Pichia*, a Member of the Core Oral Mycobiome, Exhibits Antagonism against *Candida*

**Figure 4:**
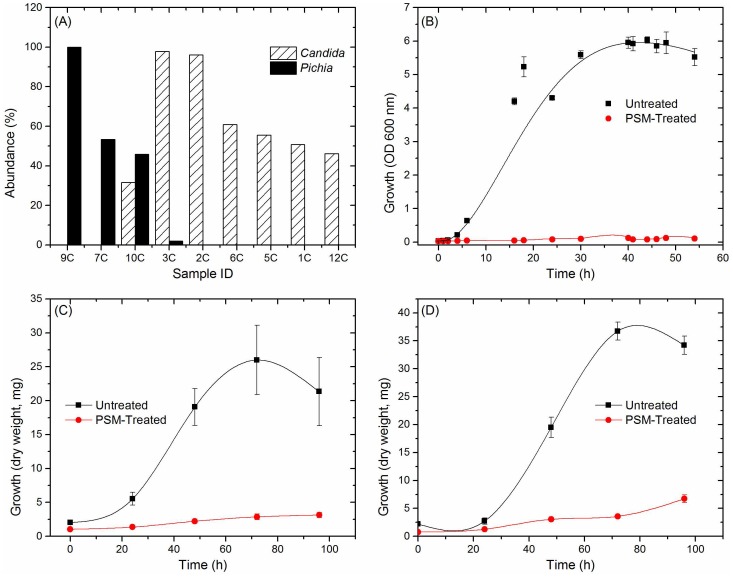
Antagonistic relation between *Pichia* and other fungi. (A) Relative abundance of *Pichia* and *Candida* in uninfected individuals. (B) Effect of PSM on growth of *Candida* was determined by measuring optical density (OD). Effect of PSM on (C) *Aspergillus*, and (D) *Fusarium* was determined by measuring dry weight of fungi. *Pichia* spent (“conditioned”) medium (PSM) was obtained by centrifuging 100-mL culture of *Pichia* grown in Sabouraud dextrose broth (SDB).

Having defined the core mycobiome, next we investigated whether members of the core oral mycobiome are associated with *Candida*, the most common oral fungal pathogen of HIV-infection [36]. Our sequencing data indicated the presence of 3 *Pichia* species, including *P. guillermondii*, *P. burtonii*, and *P. jadinii* in the tested samples. We found that decrease in *Pichia* abundance coincided with an increase in *Candida* colonization (Fig. 4A), suggesting antagonism between *Pichia* and *Candida*. Furthermore, we found that among the 4 uninfected subjects where *Pichia* was present, 24 fungal genera were absent, including *Aspergillus* and *Cryptococcus* (Table 4). In addition, in these 4 uninfected individuals, 9 fungal genera (including *Fusarium*) were present as co-colonizers (Table 4). Analysis of the abundance profile of *Fusarium* in all uninfected individuals (n = 12), revealed that its abundance was 3-fold lower when *Pichia* was present (0.016%, n = 4) compared to where *Pichia* was absent (0.048%, n = 8). Taken together, these results show that *Pichia* interacts with other fungi, with an antagonistic interaction with known pathogens including *Candida*, *Cryptococcus*, *Aspergillus* and *Fusarium*.

**Table 4:** Relationship between *Pichia* and other fungi in oral wash of uninfected patients.

Absent^1^	Co-Colonizers^2^
*Trichosporon*	*Candida*
*Epicoccum*	*Fusarium*
*Aureobasidium*	*Penicillium*
*Alternaria*	*Galactomyces*
*Aspergillus*	*Rhodotorula*
*Gibberella*	*Dioszegia*
*Corynespora*	*Albatrellus*
*Cryptococcus*	*Cladosporium*
*Clavispora*	*Cystofilobasidium*
*Glomus*	
*Yeast*	
*Lactarius*	
*Cytospora*	
*Exophiala*	
*Saccharomyces*	
*Antarctic*	
*Microbotryum*	
*Hanseniaspora*	
*Phaeosphaeria*	
*Valsa*	
*Claviceps*	
*Emericella*	
*Kodamaea*	
*Davidiella*	

1 Absent: fungi that were not detected in samples with *Pichia*.

2 Co-colonizers: fungi detected in samples where *Pichia* was present.

### *Pichia* Inhibits Growth of *Candida*, *Aspergillus* and *Fusarium*

Next, we investigated the ability of *Pichia* to inhibit growth of *C. albicans*, by allowing blastospores to grow in the presence or absence of *Pichia* spent medium (PSM). As mentioned above, our results revealed three *Pichia* species (*P. guillermondii*, *P. burtonii*, and *P. jadinii*) in the tested samples. These *Pichia* spp, along with *P. farinosa*, are common biocontrol agents used against plant pathogens [37]–[40]. Moreover, literature search showed that *P. farinosa* exhibits more biocontrol activity than *P. guilliermondii* [41]. Since *P. farinosa* and *P. guilliermondii* are very closely related to each other based on their whole genome [38] and mitochondrial DNA [42] sequences, we selected *P. farinosa* to investigate the interactions between this species and pathogenic fungi including *Candida*. As shown in Figure 4B, PSM completely inhibited *Candida* growth, demonstrating a direct inhibitory effect of *Pichia* against *Candida*. We also assessed the effect of PSM on growth of *Aspergillus* and *Fusarium* by determining their dry weight. As shown in Figure 4C and D, *Aspergillus* and *Fusarium* were unable to exhibit growth in presence of PSM. These studies demonstrated that PSM exhibits broad-spectrum activity against pathogenic fungi.

Next, we initiated studies to gain insight into the underlying mechanism for the allowing *Pichia* to inhibit *Candida*. Since *Pichia* is commonly used as a post-harvest biocontrol agent against plant pathogens [43]–[47], and several studies have suggested that this inhibitory activity involves nutrient competition, biofilm formation, germination, metabolites, secretory proteins [48]–[57], we explored whether *Pichia*-mediated inhibition of *Candida* involves these mechanisms.

### *Pichia* Overgrows *Candida* in Mixed Cultures

**Figure 5:**
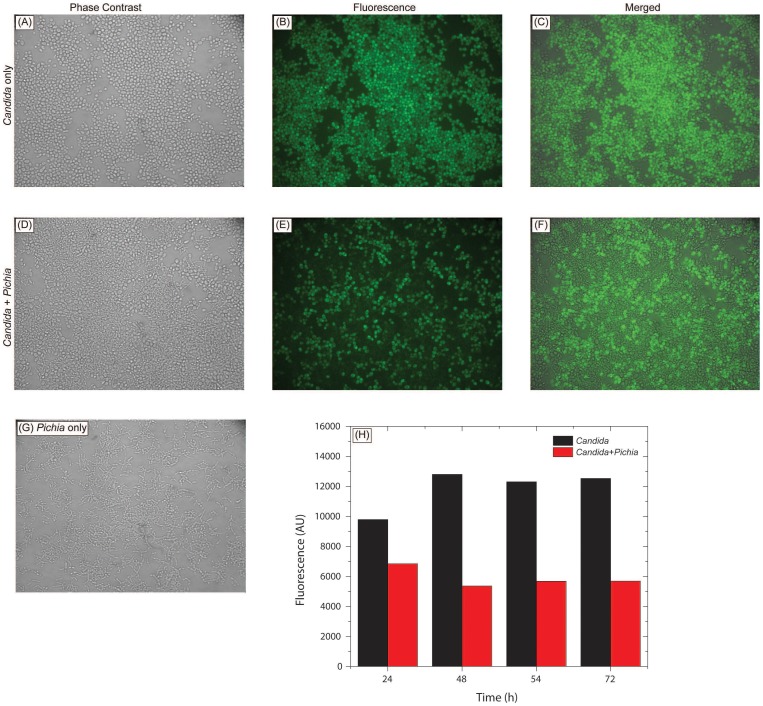
*Pichia* overgrows *Candida* in mixed cultures. *Pichia farinosa* was mixed with GFP-tagged *C. albicans* strain at 10^3^ cells each in yeast nitrogen base (YNB) growth medium and incubated at 37°C for 54 h. Growth of Candida cells was monitored by phase-contrast and fluorescence microscopy. Panels A, D, G: phase-contrast images of *Candida* alone, *Candida*+*Pichia*, and *Pichia* alone, respectively. Panels B, E: fluorescence images of *Candida* alone and *Candida*+*Pichia* mixture. Panels C, F: Composite figures with phase-contrast images merged with fluorescence image, for *Candida* only and *Candida*+*Pichia* mixture of cells. Panel H: quantitative measurement of fluorescence (excitation and emission wavelength = 485 and 435 nm, respectively).

To determine whether inhibition of *Candida* by *Pichia* is due to nutrient competition, we assessed *Candida* growth when mixed with *Pichia* at different ratios. We found that control *Candida* or *Pichia* cells exhibited robust, confluent growth when incubated by themselves (Fig. 5A–C, G). In contrast, mixing of *Candida* and *Pichia* (at 10^3^ cells each) resulted in noticeably reduced cell density (Fig. 5D–F). Quantitative measurement of fluorescence signal confirmed reduced *Candida* growth in the presence of *Pichia* cells, with up to 58% decrease in fluorescence (Fig. 5H). Similar trends were observed when *Candida* and *Pichia* cells were mixed at 10^4^ or 10^5^ cells each (data not shown). These results suggested that *Pichia* outcompetes/overgrows *Candida*.

### *Pichia* Inhibits the Ability of *C. albicans* to Form Biofilms

**Figure 6:**
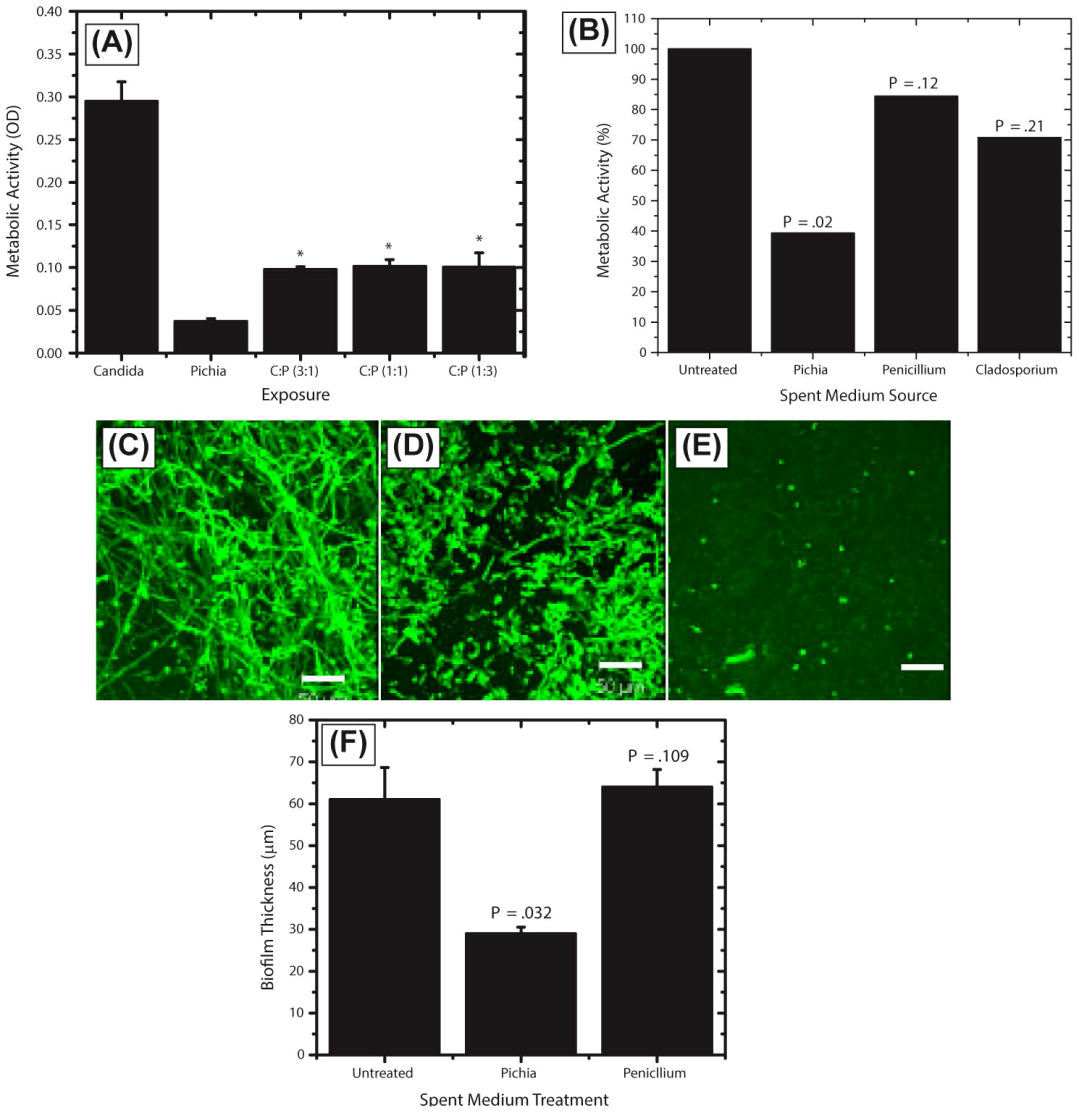
Activity of *Pichia* spent medium (PSM) against fungal biofilms. (A) Effect of *Pichia* cells on the ability of *Candida* to form biofilms. *Candida* and *Pichia* were co-incubated [*Candida*∶*Pichia* (C∶P) = 3∶1, 1∶1, or 1∶3] and biofilm formation was monitored (*P≤.002, compared to *Candida* or *Pichia* controls). (B) Effect of media supernatant obtained from *Pichia, Penicillium, or Cladosporium* on *Candida* biofilms. Mean ± SD of ≥3 separate experiments. (C–E) Confocal microscopy images of *Candida* biofilms formed in presence of (C) no media supernatant, (D) *Penicillium* supernatant or (E) *Pichia* supernatant. (F) Thickness of biofilms formed in presence of media supernatant of *Pichia* or *Penicillium*.

Our results showed that when both *Candida* and *Pichia* were present together, their abundance ratio was close to 1∶1. Therefore, to determine whether a threshold number of *Pichia* cells was necessary to inhibit *Candida* biofilms, we used mixtures of *Pichia* and *Candida* combined at different ratios (1∶1, 1∶3, 3∶1). Co-incubation of *C. albicans* with *Pichia* cells at different ratios resulted in significant inhibition of biofilm formation at all ratios tested (Fig. 6A, *P*<.05). Moreover, there was no significant difference in the extent of biofilm inhibition between the different cells densities of *Pichia* examined.

**Figure 7:**
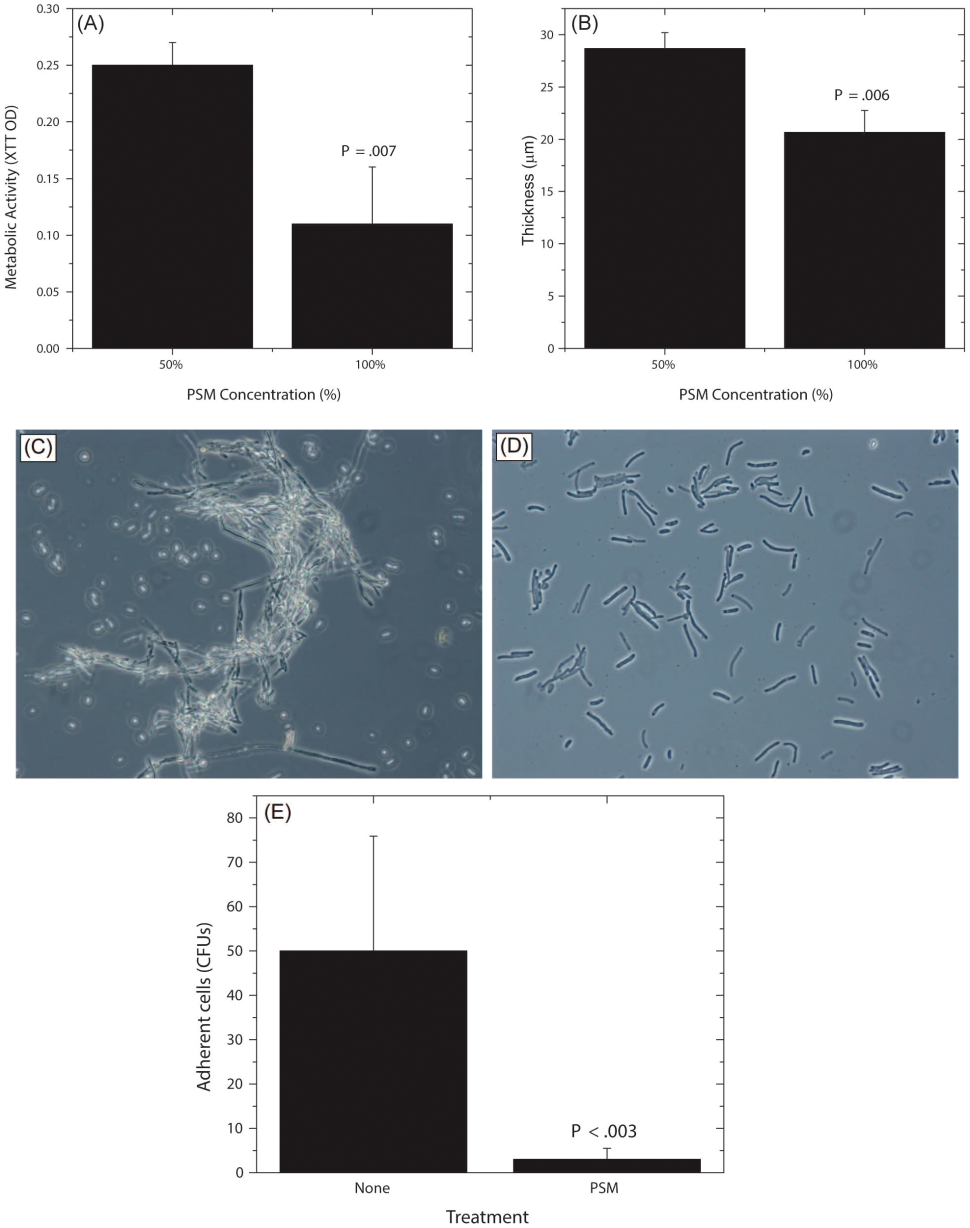
Dose dependent activity of PSM, and its effect on *Candida* germination and adhesion. Effect of undiluted and diluted (50%) PSM on (A) metabolic activity and (B) thickness of *Candida* biofilms was assessed. (C) Germination in *Candida* grown in SDB, exposed to fetal bovine serum, (D) Stunted germ tubes formed by *Candida* exposed to *Pichia* supernatant (Magnification 20X), (E) Effect of PSM on the ability of *Candida* (grown in SDB) to adhere to solid substrates. (SDB - Sabouraud dextrose broth.)

Next, to determine whether the biofilm-inhibitory activity of *P. farinosa* was mediated by secretory factor/s, we determined the effect of *Pichia* spent medium (PSM) on *C. albicans* biofilms using a metabolic activity (XTT) assay described earlier [26]. Spent medium from *Penicillium* or *Cladosporium* (both prepared similarly as PSM) was used as a control since *Penicillium* had the same abundance in HIV-infected and uninfected individuals (present in 25% of samples in each group), while *Cladosporium* was present only in uninfected participants. Our results showed that when exposed to PSM, metabolic activity of *Candida* biofilms was significantly reduced (39% compared to untreated control, *P* = .02, Fig. 6B). Moreover, exposure to equivalently prepared spent media from *Penicillium* or *Cladosporium* did not have a significant effect on *Candida* biofilms (metabolic activity = 84% and 71%, respectively, *P*≥.12 for both comparisons, Fig. 6B). Next, we used confocal laser scanning microscopy (CLSM) to determine the effect of PSM on *C. albicans* biofilm architecture. While untreated and *Penicillium*-treated *Candida* formed robust biofilms (Fig. 6C–D, respectively), exposure to PSM resulted in biofilm disruption, with sparse yeast cells and no extracellular matrix or hyphae observed (Fig. 6E). Moreover, thickness of *Candida* biofilms exposed to PSM was significantly reduced compared to that of controls (Fig. 6F, *P*<.05). Finally, to determine whether the activity of PSM is dose-dependent, we compared the ability of 50% and 100% PSM to inhibit *Candida* biofilms. Metabolic activity and thickness of *Candida* biofilms exposed to 100% PSM was significantly lower than those exposed to 50% PSM (Fig. 7A, B; *P* = .007 and .006, respectively). Taken together, these results demonstrate that the *Candida*-inhibitory activity of *Pichia* is specific and dose-dependent.

### *Pichia* Inhibits *Candida* Germination and Adhesion

Since adhesion and germination are key steps in mature *Candida* biofilm formation [58]–[60] and are known *Candida* virulence factors, we examined whether *P. farinosa* spent medium affects these processes. Our data showed that while untreated *Candida* formed robust hyphae (Fig. 7C), exposure to PSM resulted in stunted *Candida* germ tubes (Fig. 7D), indicating that a secreted component *of Pichia* inhibits *Candida* germination. We also found that the number of *Candida* colony forming units (CFUs) adhering to silicone elastomer catheter substrate when treated with *Pichia* supernatant was significantly lower than untreated *Candida* cells (Fig. 7E, *P*<.003). These results showed that *Pichia* inhibits the ability of *Candida* to germinate and adhere to catheter substrate.

### Active Ingredient of PSM Is a Protein

**Figure 8:**
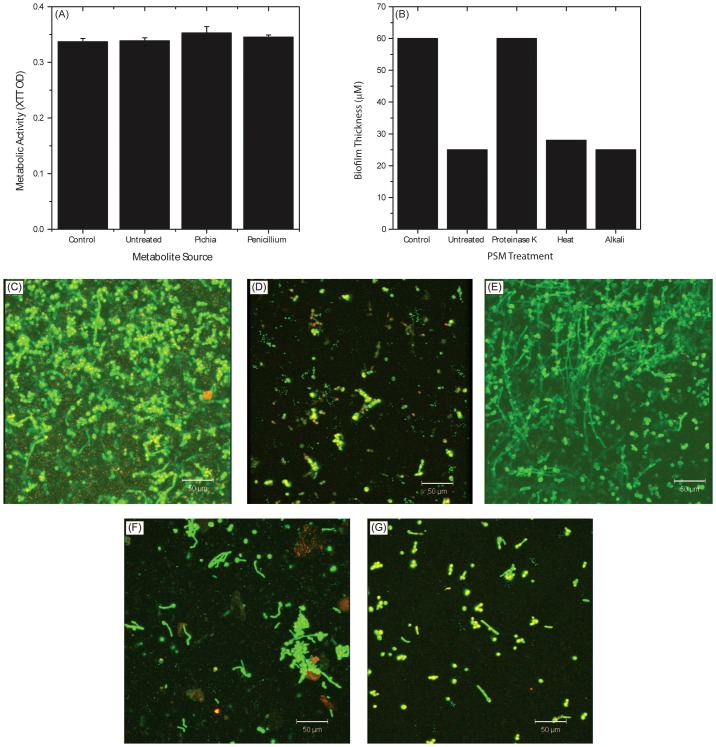
Biochemical characterization of Pichia spent medium. (A) Metabolic activity of *Candida* biofilms exposed to metabolites extracted from spent media of Pichia, Penicillium, or media control. (B) Effect of PSM exposed to proteinase, heat, or alkali on *Candida* biofilms. Confocal images show architecture of *Candida* biofilms exposed to (C) no PSM (control), (D) untreated PSM, (E) proteinase-K treated PSM, (F) heat, or (G) alkali.

To determine whether the active ingredient of PSM is a metabolite or a protein, we evaluated *Candida* growth in presence or absence of metabolites extracted [32] from PSM. Extracted *Pichia* metabolites had no effect on *Candida* growth (Fig. 8A). Next, we evaluated the influence of proteinase-, alkali-, or heat (90°C for 10 min)-treated PSM on *C. albicans* biofilms. Our data showed that proteinase-K treatment abrogated the ability of PSM to inhibit biofilms, while alkali- and heat-treatment did not have any effect, as determined by analysis of biofilm thickness (Fig. 8B) and architecture (Fig. 8C–G). These studies indicate that the active component in PSM is proteinaceous, heat-stable, and non-glycosylated.

### PSM Is Effective against Oral Candidiasis in an Experimental Murine Model

**Figure 9:**
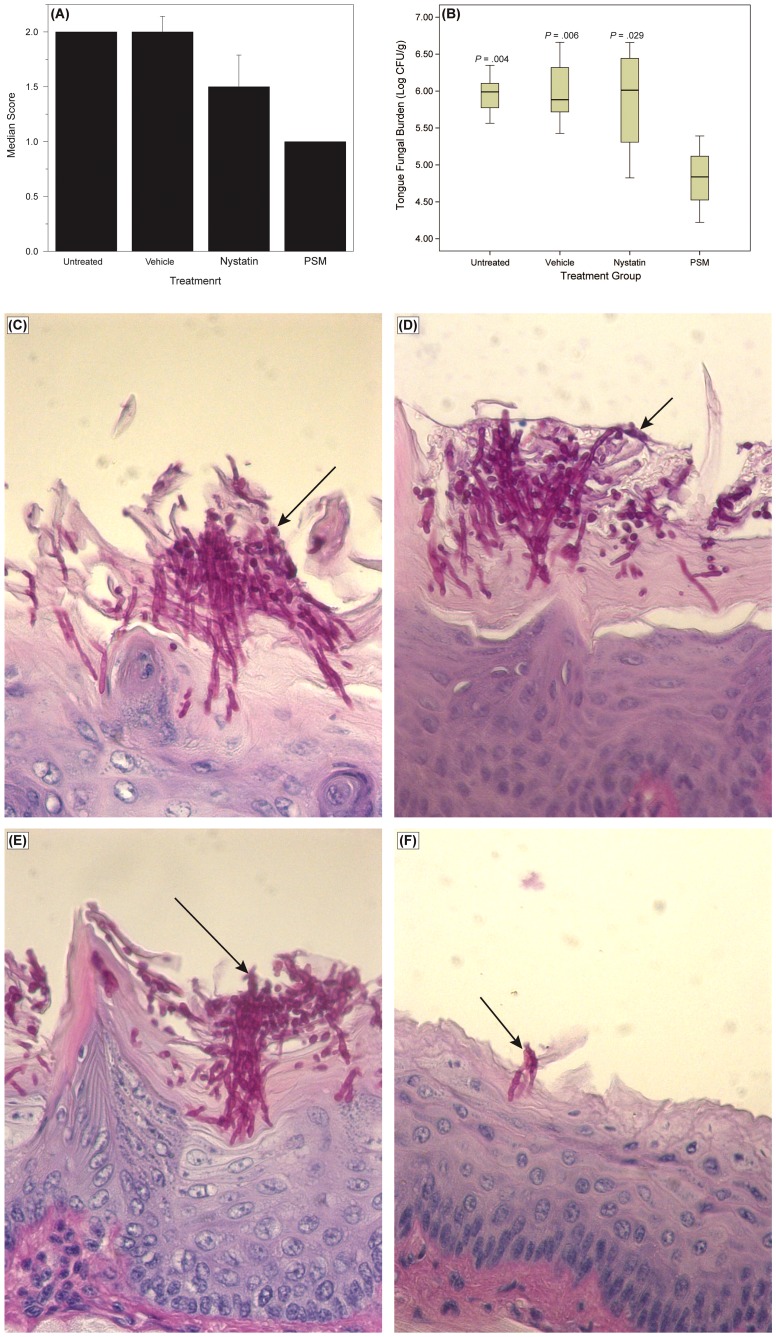
Efficacy of *Pichia* spent medium (PSM) in an experimental murine morel of oral candidiasis. Assessment of oral candidiasis in mice infected with *Candida* was performed by (A) clinical score and (B) tongue fungal burden. (A) Median clinical scores of oral candidiasis in mice after treatment with PSM and nystatin. (B) Box-plot of tissue fungal burden (log CFUs/g) in different groups of mice infected with *Candida* (*P*-values, compared to PSM treatment). Histology analyses of tissue section of tongue from mouse infected with *Candida*, followed by (C) no treatment or treated with (D) vehicle control, (E) nystatin, or (F) PSM. Arrows – fungal hyphae.

To determine whether the *in vitro* activity of PSM against *Candida* is also exhibited *in vivo*, we evaluated the efficacy of PSM in an experimental murine model of oral candidiasis. We found that at the end of treatment (Day 7), clinical score of PSM-treated mice was significant reduced compared to untreated, vehicle-treated, or nystatin-treated mice (Fig. 9A, *P* = .002 by Kruskal-Wallis test). The fungal burden of tongue from PSM-treated mice was also significantly reduced compared to untreated, vehicle-treated, or nystatin-treated controls (*P*≤.029 for all comparisons, Fig. 9B). Additionally, histological examination showed extensive tissue invasion by fungal hyphae and destruction of the epithelium in untreated or vehicle-treated controls or nystatin-treated mice (Fig. 9C–E). In contrast, tongue epithelium in PSM-treated mice revealed only superficial hyphal invasion and intact tissue structures (Fig. 9F). These results demonstrated that PSM was efficacious in treating oral candidiasis *in vivo* when tested in an experimental oral model of candidiasis.

## Discussion

In the current study, we identified the core oral mycobiome and core oral bacteriome in HIV-infected and uninfected individuals, and demonstrated that the oral mycobiome in HIV infection differs from non-diseased controls. In contrast to previous studies that characterized the bacterial component of the oral microbiome [61]–[63], our study defines both the bacterial and fungal components of the core oral microbiome in the same sample in HIV setting. Our findings revealed that the oral bacteriome of HIV-infected individuals was similar to that of uninfected individuals, indicating the presence of a shared bacteriome in these individuals. The core oral bacteriome of uninfected individuals in our study is in agreement with results reported earlier by Zaura et al. [64] who showed that 15 bacterial genera were present in the core oral bacteriome of healthy individuals.

Correlation analyses of the relationship between bacteriome and mycobiome revealed that 15 and 12 bacteria-fungi pairs were correlated significantly in samples from uninfected and HIV-infected patients, respectively. It is possible that these correlations indicate mutually dependent relationships within the oral microbiome, in which bacteria may be assisting or scavenging their fungal neighbors. Alternatively, fungal members of the microbiome may impact bacterial growth and drug susceptibility. Such interactions could influence the course and extent of oral diseases in the HIV setting. In this regard, interactions between bacteria and *Candida* have been investigated previously by Hogan and Kolter [65], who showed that *P. aeruginosa* kills hyphal form of *C. albicans* via biofilm formation. Furthermore, *Candida*-bacterial interactions are also associated with diseases like ventilator-associated pneumonia [66], [67] and bloodstream infections [68]. Our analyses revealed no correlation between *Candida* and bacteria in uninfected individuals, while in HIV-infected patients *Candida* and *Campylobacter* were negatively correlated. This correlation is in agreement with the findings of Navazesh et al. [69], who showed that antiretroviral therapy increased the risk for recovering bacteria (including *Campylobacter* species) with a concomitant decrease in the recovery rate of *Candida*, in HIV-infected women. Moreover, Workman et al. [70] reported that proteins secreted by *Campylobacter* inhibit the growth of *C. albicans*. The clinical relevance of this correlation remains to be investigated.

In the current study, we also defined the oral mycobiome of HIV-infected and uninfected study participants, and showed this fungal community to comprise up to nine different genera in both groups. To our knowledge, the only other study to have used a sequencing-based approach to identify oral fungi in HIV setting was that performed by Aas et al. [12], who analyzed sub-gingival plaque of HIV-infected patients, and reported the presence of only two fungal species (*Saccharomyces cerevisiae* and *C. albicans* in 4 and 2 patients, respectively). The difference in fungal profile between these investigators and our study may be due to differences in sample types (oral wash vs. sub-gingival plaque), detection probe (pan-fungal ITS probe vs. 18S rDNA), and sequencing technique (real-time pyrosequencing vs. rDNA sequencing).

Our study showed that the core oral mycobiome of HIV-infected patients was different from that of uninfected individuals. In a recent study, Iliev et al. [13] characterized the gut microbiome in wild type mice and isogenic *Clec7a^−/−^* mice that lack Dectin-1 (the innate immune receptor) and exhibited increased levels of chemically induced colitis. These investigators reported that there were no significant differences in bacteriome between wild type and *Clec7a^−/−^* mice, while the mycobiome profile differed between these isogenic mice, with an increase in opportunistic pathogenic fungi (*Candida* and *Trichosporon*) during colitis in *Clec7a^−/−^* mice, and a decrease in the nonpathogenic fungi (e.g. *Saccharomyces*). Thus, severe colitis in Dectin-1 knockout mice was associated with alterations in the gut mycobiome (but not the bacteriome). This study is similar to our findings, showing that HIV disease is associated with changes in the oral mycobiome, and highlight the importance of characterizing the mycobiome and its role in disease.

Our analyses showed that increase in *Candida* colonization was associated with a concomitant decrease in the abundance of *Pichia*, suggesting an antagonistic relation between these two fungi. In addition, these analyses also suggested that *Pichia* exhibits antagonistic interaction with other known pathogens including *Cryptococcus*, *Aspergillus* and *Fusarium*. These interactions were confirmed using growth assays that demonstrated broad-spectrum inhibitory activity of *Pichia*. The anti-*Candida* activity of *Pichia* was validated in vivo using an experimental murine model of oral candidiasis.

The biocontrol activity of *Pichia* against fungal plant pathogens has been shown to involve multiple mechanisms, including biofilm formation and germination [48]–[53]. The results of the current study are in agreement with these findings, where we showed that the anti-*Candida* activity of *Pichia* is mediated by inhibition of *Candida* growth and virulence factors like germination, adherence, and biofilm formation. It is well known that interfering with virulence factors decreases the ability of *Candida* to cause infection including oral candidiasis [71]–[79]. Therefore, *Pichia*, by inhibiting *Candida* virulence factors may limit the ability of this pathogenic fungus to cause infection. Such involvement of virulence factors is a common theme in interactions among microorganisms in the context of human infection [65].

*Pichia* biocontrol activity has also been attributed to nutrient limitation [39], [41], [51], [80]. Our results showed that *Pichia* outcompetes *Candida* when the two fungi are mixed together and allowed to grow. The possible reason for this phenomenon could be due to the ability of *Pichia* to consume nutrients more efficiently than *Candida*. Alternatively, it is possible that *Pichia* secretes factor/s that attenuate the ability of *Candida* to grow. Data in support of the latter possibility can be derived from our findings that *Pichia* spent medium inhibits pathogenic fungi including *Candida*, *Aspergillus*, *Fusarium*, and *Cryptococcus*. Furthermore, our results also demonstrate that the inhibitory activity of PSM is proteinaceous in nature, and not a metabolite. Earlier studies have shown that biocontrol activity of *Pichia* against fungal plant pathogens is mediated by secretory metabolite or proteins [48], [53], [81], [82].

In conclusion, we identified the core bacteriome and mycobiome in HIV setting, and identified HIV-specific changes in the mycobiome. We also identified a critical antagonistic interaction between *Pichia* and fungal pathogens including *Candida*. This interaction was demonstrated using in vitro and in vivo models. We also defined the mechanisms underlying the antagonistic interaction between *Pichia* and *Candida*. Our findings show for the first time that normal fungal community interacts with *Candida* in the oral cavity. Detailed investigations are warranted to purify and characterize the secretory factor/s mediating such interactions, and their mechanism/s of action at the molecular level. Our findings have wide implications regarding the discovery of novel antifungal agents that new therapeutic approaches for the management of fungal infections.

## Supporting Information

Method S1 Additional details of PCR, pyrosequencing and correlation analyses.
(DOCX)

Table S1 Relative abundance of bacteria in oral wash of HIV-infected and uninfected study participants.
(XLS)

Table S2 Relative abundance of fungi in oral wash of HIV-infected and uninfected study participants.
(XLS)
