# Supplementary material for: Oral Mycobiome Analysis of HIV-Infected Patients: Identification of Pichia as an Antagonist of Opportunistic Fungi
Source: PLoS Pathog. 2014 Mar 13;10(3):e1003996. doi: 10.1371/journal.ppat.1003996 (PMC3953492; doi:10.1371/journal.ppat.1003996)
Supplement: Method S1 — Additional details of PCR, pyrosequencing and correlation analyses. (DOCX) [file ppat.1003996.s001.docx]

**SUPPORTING METHODS**

**PCR analysis:** PCR reactions were carried out on approximately 10 ng template DNA in a 20-µl (final volume) reaction mixture consisting of 1 X PCR buffer, 0.01% bovine serum albumin, 2.5 mM MgCl_2_, each dNTP at a concentration of 0.25 mM, each primer at a concentration of 0.5 µM, and 0.5 U of AmpliTaq Gold DNA polymerase (ABI, Foster City, CA). Initial denaturation at 94 °C for 11 min was followed by 35 cycles of denaturation for 30s each at 94 °C, annealing at 50 °C for 30 s, and progressive extension at 72 °C for 2 min. Following the 35 cycles there was a final extension time of 30 min to minimize artifacts induced by TAQ polymerase. Fungal PCR products were separated on the SCE 9610 capillary DNA sequencer (Spectrumedix LLC, State College, PA) using GenoSpectrum software to convert fluorescent output into electropherograms. Relative peak abundance of fungal amplicons was calculated by dividing individual peak heights by the total peak heights in a given electropherogram using a custom PERL script. Interleaved, normalized abundances were compared as stacked histograms using Microsoft Excel. Mean normalized abundance for each amplicon was calculated from the three PCR replicates of each sample, excluding means below 1%. Results were analyzed by visual inspection and Principal Coordinate (PCO) analysis using Multivariate Statistical Package (MVSP, Kovach Computing Services, Wales, UK). Normalized abundance of each peak in the electropherogram was calculated with respect to the total peak area, since it is not possible to calculate absolute abundances with either the LH-PCR or MTPS technology.

**Multitag Pyrosequencing Analysis:** Microbiome analysis was performed using multitag 454 pyrosequencing (MTPS) technique, which was used for detailed characterization of nucleic acids and has the advantages of accuracy, flexibility, parallel processing, and easy automation potential [[1](#_ENREF_1)]. We generated a set of 24 emulsion PCR fusion primers that contains the 454-emulsion PCR adapter, joined to a 7 base “barcode” along with the appropriate target primers. The generated MTPS data were demultiplexed using a custom PERL script and analyzed us BLAST and GenBank. The annotations for each sequence were organized by taxa, which were then expressed as percentage of the total oral community in each sample. Fungal and bacterial sequences were compared with the online database using the BLAST interface of Web Accessible Sequence Analysis for Biological Inference (WASABI) as well as against the NCBI nucleotide database. Taxa with < 1% abundance were assigned a 0 value, and not included in the analysis. The microbiome abundance data was divided into independent mycobiome and bacteriome data matrices and correlation analysis was conducted as described in Supplemental Methods (online).

Specifically for this experiment, we used the A Adapter with a barcode and the ITS1F sequence for the forward primer and the B Adapter with the ITS4A sequence (i.e. without a tag) for the reverse primer. All sequences were read from the A Adapter side. Similar approach was used for the 16S-based sequencing. Thus, each oral rinse sample was amplified with a uniquely barcoded set of forward and reverse rRNA primers and then up to 24 samples were pooled and subjected to emulsion PCR and pyrosequenced using a GS-FLX pyrosequencer. Data from each pooled sample were “deconvoluted” by sorting the sequences into bins based on the barcodes using custom PERL scripts. Thus, we were able to normalize each sample by the total number of reads from each barcode. Several groups have subsequently employed various barcoding strategies to analyze multiple samples and this strategy is now well accepted [[2-5](#_ENREF_2)].

**Correlation Analysis:** The microbiome abundance data was divided into independent mycobiome and bacteriome data matrices and correlation analysis was conducted using R statistical computing software (CRAN mirror- version 2.13.2). The “ Psych” package was used to correlate the matrices using the “*corr.test*” function, which reveals the coefficients as well as the statistics for each independent test. The correlation analysis was performed using pairwise Spearman’s correlation and two-tailed probability of *t* for each correlation. The function “*Circle.corr*” (http://gallery.r-enthusiasts.com/graph/Correlation_matrix_circles,152) was used to graphically illustrate the correlation coefficients and significant correlations (p < .05). The circle graph plots the correlation coefficients using the diameter of the circle to represent the absolute value of each correlation test and the red and blue shades represent the positive and negative correlations, respectively.

**REFERENCES CITED IN SUPPORTING METHODS**

1. Gillevet PM (2006) Inventor BioSpherex LLC, assignee. Multitag Sequencing and Ecogenomic Analysis. Patent Cooperative Treaty.

2. Parameswaran P, Jalili R, Tao L, Shokralla S, Gharizadeh B, et al. (2007) A pyrosequencing-tailored nucleotide barcode design unveils opportunities for large-scale sample multiplexing. Nucleic Acids Res 35: e130.

3. Hoffmann C, Minkah N, Leipzig J, Wang G, Arens MQ, et al. (2007) DNA bar coding and pyrosequencing to identify rare HIV drug resistance mutations. Nucleic Acids Res 35: e91.

4. Turnbaugh PJ, Hamady M, Yatsunenko T, Cantarel BL, Duncan A, et al. (2009) A core gut microbiome in obese and lean twins. Nature 457: 480-484.

5. Binladen J, Gilbert MT, Bollback JP, Panitz F, Bendixen C, et al. (2007) The use of coded PCR primers enables high-throughput sequencing of multiple homolog amplification products by 454 parallel sequencing. PLoS ONE 2: e197.
